# A long-term survivor of Bland-White-Garland syndrome with systemic collateral supply: A case report and review of the literature

**DOI:** 10.1186/1471-2482-5-23

**Published:** 2005-12-15

**Authors:** N Barbetakis, A Efstathiou, N Efstathiou, P Papagiannopoulou, V Soulountsi, I Fessatidis

**Affiliations:** 1 Cardiothoracic Surgery Department, Geniki Kliniki, Thessaloniki, Greece; 2 Cardiology Department, Geniki Kliniki, Thessaloniki, Greece; 3 Intensive Care Unit, Geniki Kliniki, Thessaloniki, Greece

## Abstract

**Background:**

land-White-Garland syndrome (anomalous origin of the left coronary artery from the pulmonary artery) is a rare disease which may result in myocardial infarction, congestive heart failure and sometimes death during the early infantile period. Case presentation: A succesfully treated case of a 45-year-old mother of 2 children with Bland-White-Garland syndrome and concomitant severe mitral regurgitation is presented. Subsequent therapy consisted of ligation of the anomalous origin of the left coronary artery, anastomosis of the left internal mammary artery to the left anterior descending branch and mitral valve replacement. Continuous blood flow from the left coronary artery ostium during extracorporeal circulation and aorta clamping suggested systemic collateral supply. Conclusions: Recognition and diagnosis of Bland-White-Garland syndrome is important due to its potentially life-threatening complications.

## Background

Anomalous origin of the left coronary artery arising from the pulmonary artery (Bland-White-Garland syndrome also known as ALCAPA syndrome), is a rare congenital abnormality affecting 1 in 300.000 live births, accounting for 0,5% of cases of congenital heart disease [1].

Patients with Bland-White-Garland (BWG) syndrome who survive past childhood often have varying symptoms of myocardial ischaemia, impaired left ventricular function, mitral regurgitation and progressive heart failure, depending on the development of collateral circulation. The majority of patients die in infancy [2].

A case of a 45-year-old woman referred to our institution with the diagnosis of mitral regurgitation is presented. Detailed diagnostics revealed BWG syndrome. Subsequent therapy consisted of ligation of the anomalous origin of the left coronary artery, anastomosis of left internal mammary artery (LIMA) to the left anterior descending (LAD) branch and mitral valve replacement with a mechanical prosthesis.

## Case presentation

A 45-year-old mother of 2 children was referred to our hospital because of progressive exertion dyspnea, fatigue and hemoptysis. Her pregnancies and deliveries had been normal. The patient was known to have had a heart murmur since the age of 15 years. Physical examination revealed a regular pulse of 72 beats per minute, blood pressure of 120/70 mm Hg and a pansystolic murmur at the apex. There were no clinical signs of left or right heart failure.

**Figure 1:**
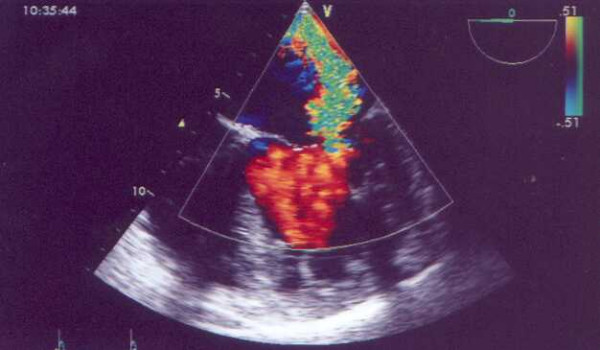
Severe mitral regurgitation.

**Figure 2:**
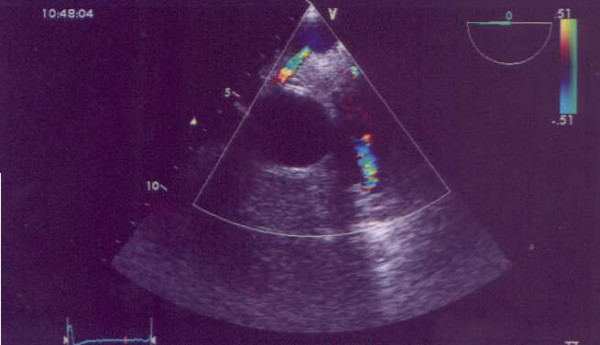
Possible communication between left coronary artery and pulmonary artery.

Electrocardiogram showed lateral repolarisation abnormalities, left axis deviation and poor R-wave progression (V1-V3). Chest X-ray revealed cardiomegaly (cardiothoracic ratio: 60%). Transthoracic echocardiography demonstrated a mildly dilated left ventricle with satisfactory contraction (ejection fraction: 60%) and severe mitral regurgitation (Figure 1). A transesophageal echocardiogram showed a dilated right coronary artery originating from right coronary cusp. The origin of the left coronary artery in the left coronary cusp could not be found. A possible communication between left coronary artery and pulmonary artery was also detected but it was not possible to evaluate the direction of flow (Figure 2).

Coronary angiography demonstrated a dilated right coronary artery to arise from the appropriate sinus of Valsalva but the left coronary artery could not be identified. The left coronary artery and the pulmonary trunk were supplied by collaterals.

Saturation sampling during right heart catheterization showed a mild left to right shunt (Qp/Qs = 1,6/1) with a clear saturation gap of 15% between the lower and the higher section of the pulmonary trunk (64,2 % and 79,3% respectively). Pulmonary hypertension was also confirmed (systolic pressure: 61 mm Hg, diastolic pressure: 22 mm Hg). The patient was qualified for surgical correction.

A LIMA to LAD anastomosis was carried out initially. Circumflex or obtuse marginal arteries were not graftable because of small calibre. After establishing extracorporeal circulation and clamping the aorta the heart was arrested using cold blood cardioplegic solution. The pulmonary arteriotomy revealed the ostium of left coronary artery on the right posterolateral wall of the pulmonary trunk, 0,5 cm above the posterior commissure of pulmonary valve. Continuous blood outflow from the ostium of the left coronary artery was observed during the procedure, despite cardioplegia and lack of coronary circulation, indicating systemic blood supply to the left coronary artery. Ligation of the anomalous origin of the left coronary artery was performed by means of a purse string polypropylene suture with venous pledgets.

Mitral valve was found to be thin and flimsy and repairing it, was not feasible. Replacement was carried out utilizing mechanical prosthesis. Posterior leaflet was retained. The excised valve showed myxomatous degeneration pathology.

Weaning from cardiopulmonary bypass was uneventful and immediate spontaneous restoration of rhythmic heart function with stable sinus rhythm was observed. Postoperative period was straightforward. Three months later, patient's dyspnea had improved by one NYHA class with a great improvement in exercise tolerance.

## Conclusion

Bland, White and Garland first reported the association of anomalous origin of left coronary artery from pulmonary artery with a hypertrophied left ventricle and attacks of dyspnea, pallor and profuse sweating in a boy who died at the age of 3,5 months [3]. The estimated incidence of BWG syndrome is 1/300.000 live births (between 0,24% and 0,46% of all congenital cardiac anomalies). Some believe that this is a significant underestimation of the true incidence, as many patients may be asymptomatic until their death and therefore remain undiagnosed [4]. BWG syndrome develops before birth when the systemic and pulmonary arterial pressures are equal and there is antegrade flow in both the left and right coronary artery. In the neonatal period, this gradually changes as the pulmonary blood pressure diminishes, the ductus arteriosus closes and the flow in the left coronary artery reverses. The development of collateral circulation between the right and left coronary artery during closure of the duct and lowering of the pulmonary pressure will determine the extent of myocardial ischemia. Patients with well established collaterals have been classified as the "adult type" and those with no collaterals as the "infantile type". Edwards believes that these functional states, actually represent different phases in collateral circulation and the changes engendered by each patient determine his clinical course[5]. Rarely, patients may survive to the sixth or seventh decades of life [6].

Onset of symptoms is usually observed in the neonatal period. Beginning at 1 to 3 months, feeding or crying induces dyspnea, profuse sweating, pallor, fatigue and a semblance of pain. Between attacks, physical examination is frequently normal. Signs of heart failure and failure to thrive are common. However, cases of totally asymptomatic adult patients have been reported [7,8]. Our patient had no symptoms until the age of 44 years and had 2 uncomplicated pregnancies and deliveries. Nightingale et al and Maeder et al similarly reported three patients with uneventful pregnancies [9,10]. It can be presumed that the asymptomatic course of our patient was due to an optimal balance between sufficient collateral flow from the right to left coronary arteries, a minimal coronary steal from the pulmonary artery and a systemic blood supply to the left coronary artery. The possibility of atherosclerosis of the collateral circulation could be a reason why this patient at the age of 45 years became symptomatic. There was no proof for this systemic collateral supply but the continuous outflow of the blood from the left coronary ostium during extracorporeal circulation is a strong evidence. Magnetic resonance imaging and angiography were recommended to the patient postoperatively but she declined. There are three similar cases describing BWG syndrome with a possible systemic collateral supply [11-13]. Only in one of them, angiography confirmed systemic blood supply from a bronchial artery.

Electrocardiographic abnormalities of BWG syndrome include left axis deviation, abnormal Q-waves in leads I and aVL, poor R-wave progression resembling lateral myocardial infarction and left ventricular hypertrophy (with or without ST-segment depression). In theory, the more extensive the coronary collaterals, the better preserved the myocardial function due to lack of ischemia, although the degree of coronary steal may then become significant.

Pulmonary hypertension gradually develops because of combined left-to-right shunting, left ventricular dysfunction and mitral regurgitation. Mitral regurgitation is thought to result from the dysfunction of the ischemic papillary muscles and adjacent myocardium. Rarely, mitral regurgitation is due to prolapse or an associated lesion such as mitral cleft or short chordae [14]. In our case severe regurgitation was due more to myxomatous degeneration of the mitral valve than ischaemic mitral regurgitation, as indicated by the pathological findings of the excised valve.

Surgery is recommended in patients with BWG syndrome, even in the absence of symptoms or a significant left-to-right shunt syndrome, given the risk of ventricular arrhythmias and sudden death [15]. Whenever possible and especially in children, a 2-coronary system will be established by the direct implantation of the left coronary artery into the ascending aorta. Alternative options more preferable in adult patients include the ligation of the left coronary artery ostium combined with aortocoronary bypass grafting using venous grafts, the internal mammary artery or the radial artery [16]. Reimplantation is technically difficult in adults because of stretching on the coronary repair and the friable nature of the dilated coronary artery. A simplified technique has recently described by Laks et al [17]. According to this technique the left coronary artery and a cuff of pulmonary artery wall are excised as a button and transferred to the aorta. The defect in the pulmonary artery is replaced with a pericardial patch. The need to clamp the aorta in an ischemic myocardium probably contributes to the mortality with this procedure. The intrapulmonary technique designed by Takeuchi et al is another solution but has the potential disadvantages of pulmonary artery stenosis, aortic regurgitation or tunnel stenosis [18]. In our case an off pump LIMA to LAD anastomosis was carried out initially, followed by ligation of the anomalous origin of the left coronary artery and mitral valve replacement. It is very difficult to say whether the patient's clinical improvement was due to the mitral valve replacement or correction of the coronary artery abnormality or both.

Although BWG syndrome is a rare condition presenting in adulthood, awareness of this congenital abnormality is important, since early diagnosis and treatment may prevent irreversible damage to the myocardium and subsequent complications including myocardial infarction, heart failure, mitral regurgitation and sudden death. Certain echocardiographic findings are highly suggestive of the syndrome, including the demonstration of a dilated right coronary artery arising from the aorta, a continuous blood flow from the left coronary artery into the pulmonary artery and visualisation of dilated interventricular septal collaterals. Diagnosis usually established by a coronary angiogram, which shows a dilated and tortuous right coronary artery with collateral filling of the left coronary system. Nuclear magnetic resonance imaging is also able to locate the origin of left coronary artery.

The present case illustrates that BWG syndrome can be asymptomatic over a long time, even during pregnancy. Systemic collateral blood supply to the coronaries may be the cause for the asymptomatic course and patient's survival.

## Abbreviations

BWG: Bland-White-Garland

LIMA: Left internal mammary artery

LAD: Left anterior descending branch

## Competing interests

The author(s) declare that they have no competing interests

## Authors' contributions

All authors took part in the care of the patient and contributed equally in carrying out the medical literature search and preparation of the manuscript. All authors have approved the final manuscript.

## Pre-publication history

The pre-publication history for this paper can be accessed here:



## References

[B1] Perloff JK, Perloff JK (1994). Anomalous origin of the left coronary artery from the pulmonary trunk. the clinical recognition of congenital heart disease.

[B2] Wesselhoeft H, Fawcett JS, Johnson AL (1968). Anomalous origin of the left coronary artery from the pulmonary trunk. Its clinical spectrum, pathology and pathophysiology based on a review of 140 cases with seven further cases. Circulation.

[B3] Bland EF, White PD, Garland J (1933). Congenital anomalies of the coronary arteries: report of an unusual case associated with cardiac hypertrophy. Am Heart J.

[B4] Wollenek G, Damanig E, Salzer-Mufar U (1993). Anomalous origin of the left coronary artery: a review of surgical management in 13 patients. J Cardiovasc Surg.

[B5] Edwards JE (1964). The direction of blood flow in coronary arteries arising from the pulmonary trunk (editorial). Circulation.

[B6] Purut CM, Sabiston DC (1991). Origin of the left coronary artery from the pulmonary artery in older adults. J Thorac Cardiovasc Surg.

[B7] Arsan S, Naseri E, Keser N (1999). An adult case of Bland-White-Garland syndrome with huge coronary artery aneurysm. Ann Thorac Surg.

[B8] Masciolli G, Turelli A, Niccoli L (1993). Anomalous origin of the left coronary artery from the pulmonary artery. A rare case diagnosed in adulthood. G Ital Cardiol.

[B9] Nightingale AK, Burell CJ, Marshall AJ (1998). Anomalous origin of the left coronary artery from the pulmonary artery: natural history and normal pregnancies. Heart.

[B10] Maeder M, Vogt P, Ammann P, Rickli H (2004). Bland-White-Garland syndrome in a 39-year-old mother. Ann Thorac Surg.

[B11] Ishihata T, Takeda H, Katohno E (1994). An adult case of Bland-White-Garland syndrome with collaterals from bronchial artery. Heart Vessels.

[B12] Yuda S, Nakatani S, Kouyama K (1997). Evaluation of intramyocardial coronary flow velocity pattern before and after surgical repair of Bland-White-Garland syndrome by pulsed doppler echocardiography. J Cardiol.

[B13] Karolczak MA, Wieteska J, Bec L, Madry W (2001). Anomalous origin of the left coronary artery (LCA) from the pulmonary trunk (Bland-White-Garland syndrome) with systemic collateral supply. Med Sci Monit.

[B14] Laborde F, Marchand M, Leca F, Jarreau MM, Dequirot A, Hazan E (1981). Surgical treatment of anomalous origin of the left coronary artery in infancy and childhood. Early and late results in 20 consecutive cases. J Thorac Cardiovasc Surg.

[B15] Alexi-Meskishvili V, Berger F, Weng Y, Lange PE, Hetzer R (1995). Anomalous origin of the left coronary artery from the pulmonary artery in adults. J Card Surg.

[B16] Backer CL, Stout MJ, Zales VR (1992). Anomalous origin of the left coronary artery. A twenty-year review of surgical management. J Thorac Cardiovasc Surg.

[B17] Laks H, Ardehali A, Grant PW, Allada W (1995). Aortic implantation of anomalous left coronary artery: an improved surgical approach. J Thorac Cardiovasc Surg.

[B18] Takeuchi S, Imamura H, Katsumoto J (1979). New surgical method for repair of anomalous left coronary artery from the pulmonary artery. J Thorac Cardiovasc Surg.

